# Studies on Anticonvulsant Effects of Novel Histamine H3R Antagonists in Electrically and Chemically Induced Seizures in Rats

**DOI:** 10.3390/ijms19113386

**Published:** 2018-10-29

**Authors:** Alaa Alachkar, Dorota Łażewska, Gniewomir Latacz, Annika Frank, Agata Siwek, Annamaria Lubelska, Ewelina Honkisz-Orzechowska, Jadwiga Handzlik, Holger Stark, Katarzyna Kieć-Kononowicz, Bassem Sadek

**Affiliations:** 1 Department of Pharmacology & Therapeutics, College of Medicine & Health Sciences, United Arab Emirates University, P.O. Box 17666, Al Ain, UAE; 201590025@uaeu.ac.ae; 2 Department of Technology and Biotechnology of Drugs, Faculty of Pharmacy, Jagiellonian University Medical College, 9 Medyczna Street, 30-688 Kraków, Poland; dlazewska@cm-uj.krakow.pl (D.Ł.); glatacz@cm-uj.krakow.pl (G.L.); annamaria@outlook.com (A.L.); ewelina.honkisz@uj.edu.pl (E.H.-O.); jhandz@interia.pl (J.H.); mfkonono@cyf-kr.edu.pl (K.K.-K.); 3 Institute of Pharmaceutical and Medicinal Chemistry, Heinrich Heine University Düsseldorf, Universitaetsstr. 1, 40225 Düsseldorf, Germany; a.frank@hhu.de (A.F.); stark@hhu.de (H.S.); 4 Department of Pharmacobiology, Faculty of Pharmacy, Jagiellonian University Medical College, 9 Medyczna Street, 30-688 Kraków, Poland; agat.siwek@uj.edu.pl

**Keywords:** histamine H3 receptors, antagonists, anticonvulsant, maximal electroshock, pentylenetetrazole, strychnine, antiproliferative action, metabolic stability

## Abstract

A newly developed series of non-imidazole histamine H3 receptor (H3R) antagonists (**1**–**16**) was evaluated in vivo for anticonvulsant effects in three different seizure models in Wistar rats. Among the novel H3R antagonists examined, H3R antagonist **4** shortened the duration of tonic hind limb extension (THLE) in a dose-dependent fashion in the maximal electroshock (MES)-induced seizure and offered full protection against pentylenetetrazole (PTZ)-induced generalized tonic-clonic seizure (GTCS), following acute systemic administration (2.5, 5, 10, and 15 mg/kg, i.p.). However, only H3R antagonist **13,** without appreciable protective effects in MES- and PTZ-induced seizure, fully protected animals in the strychnine (STR)-induced GTCS following acute systemic pretreatment (10 mg/kg, i.p.). Moreover, the protective effect observed with H3R antagonist **4** in MES-induced seizure was completely abolished when animals were co-administered with the H3R agonist (*R*)-α-methylhistamine (RAMH, 10 mg/kg, i.p.). However, RAMH failed to abolish the full protection provided by the H3R antagonist **4** in PTZ-induced seizure and H3R antagonist **13** in STR-induced seizure. Furthermore, in vitro antiproliferative effects or possible metabolic interactions could not be observed for compound **4**. Additionally, the predictive in silico, as well as in vitro, metabolic stability for the most promising H3R antagonist **4** was assessed. The obtained results show prospective effects of non-imidazole H3R antagonists as innovative antiepileptic drugs (AEDs) for potential single use against epilepsy.

## 1. Introduction

Epilepsy is considered as a brain disease characterized by repeated seizures due to different complex causes including idiopathic (ion channel or protein defects), symptomatic stroke, head injury (tumors in the brain or central infectious diseases), metabolic disorders (e.g., imbalance of several electrolytes, uremia, and hyperglycemia), or genetic mutation of DNA sequences in genes responsible for encoding various ion channels or neurotransmitter receptors [1,2]. In addition, many patients diagnosed with epilepsy also show cognitive comorbid features, for example, deficiency in intellectual ability; impaired learning and memory; and behavioral neuropsychiatric disorders, such as depression, aggression, or problems in social relationships that intensify the costs for their healthcare [3]. Clinically obtainable antiepileptic drugs (AEDs) still have a large number of non-responders with the clinical drawback that around 30% of epileptic patients do not respond to current therapies [4,5]. More than fifteen new AEDs, including lamotrigine, levetiracetam, retigabine, and perampanel, were approved during the last twenty-five years [6]. However, numerous AEDs cause several unwanted adverse effects, with the most common ones being behavioral and cognitive deficiencies, weight gain, coordination problems, dizziness, and gait disturbance [6]. As long life drug treatment is indispensable, the search for new and more effective AEDs with improved safety profiles is an imperative therapeutic goal [5]. Therefore, there is still a high therapeutic need for new medical entities.

Preclinical and clinical studies indicated that several brain monoamines such as serotonin, dopamine, noradrenaline, histamine, or melatonin play a significant role in seizure activity [7,8]. Previous studies have investigated in the brain histamine’s role in seizure pathophysiology [9,10]. Accordingly, histamine itself is considered as an endogenous anticonvulsant [9,11], while histamine H1 receptor (H1R) antagonists were found to prompt or promote seizures [12,13]. Furthermore, preclinical experiments in several rodents revealed that inhibition of *N*-methyltransferase, the histamine metabolizing enzyme in the central nervous system (CNS), using metoprine elevated the levels of brain histamine and, therefore, reduced seizure susceptibility [14,15,16,17]. On the other hand, α-fluoromethylhistidine (α-FMH, the histamine synthesizing enzyme in the CNS) escalated seizure activity in experimental animals as a result of inhibition of histidine decarboxylase (HDC), which minimized the biosynthesis of brain histamine [18].

Histamine acts by four distinct histamine receptor subtypes (H1R–H4R), which belong to class A of the G-protein coupled receptor family [19]. H1R and H2R are largely found in the CNS and periphery, H3Rs are abundant in the brain, while H4Rs are chiefly expressed in mast cells and leukocytes [20]. Activation of H1R and H2R stimulates slow excitatory postsynaptic potential, while H3Rs is coupled to G_i_/G_o_-proteins, which regulate the biosynthesis and release of brain histamine by acting as auto-receptors [19,21,22]. Moreover, H3Rs operating as hetero-receptors can also modulate the release of various other brain neurotransmitters in different brain regions, for example, acetylcholine, glutamate, gamma-aminobutyric acid (GABA), norepinephrine, serotonin, and dopamine [23].

Previous experimental observations from our research [24,25,26,27,28,29,30] and that of other research groups [18,31,32,33,34] signified the anticonvulsant activity of several histamine H3R antagonists, which could be connected to their antagonistic effects on histamine H3Rs. In different rodent models of seizure, both the imidazole-based H3R antagonist thioperamide [32,35,36] and non-imidazole-based H3R antagonists [26,27,28,29] reduced seizures or displayed full protective effects in the maximal electroshock (MES)- and pentylenetetrazole (PTZ)-induced seizure models. Pitolisant (PIT), which is the first H3R antagonist to reach the market (Wakix^®^; Bioprojet Pharma—as an orphan drug for the treatment of narcolepsy [37]), also presented effectiveness in different animal models of epilepsy [25,33,38], which could not be confirmed so far in clinical trials [34] (Table 1).

Based on the aforementioned preclinical and clinical results, the current study examined the anticonvulsant effects of newly developed *N*-alkyl-substituted (homo)piperidine ether derivatives (**1**–**16**) (Table 1). These novel H3R antagonists (**1**–**16**) were developed as structural modifications of PIT and comprise different cycloalkylamine functionalities in the basic part of target ligands, different alkyl chain lengths (five or six methylene groups), as well as variable positions of methyl at the basic piperidine ring (3-CH_3_ or 4-CH_3_) (Table 1). First, their in vitro affinities on human histamine H3Rs (*h*H3Rs) were evaluated. Second, their anticonvulsant effects were examined in the MES-, PTZ-, and strychnine (STR)-induced seizures in male adult rats. The latter in vivo seizure test models were selected based on earlier preclinical observations, in which the anticonvulsant efficacy of numerous imidazole as well as non-imidazole H3R antagonists was established [39,40,41]. Third, the ability of the CNS penetrant H3R agonist (*R*)-α-methylhistamine (RAMH) to counteract the protection provided by the most promising H3R antagonists was considered. Fourth, the in vitro selectivity profile towards HRs subtypes for compounds with the most promising anticonvulsant effects was evaluated on human HRs expressed in different cell lines. Fifth, the absorption-distribution-metabolism-excretion-Toxicology (ADME-Tox) profile of the H3R antagonist with the most promising anticonvulsant efficacy was further assessed for antiproliferative activity, for metabolic stability, and for the potential of drug-drug interaction.

## 2. Material and Methods

### 2.1. In Vitro Pharmacology

#### 2.1.1. Human Histamine H3 Receptor (hH3R) Binding Affinity for Tested Compounds **1**–**16**

As described previously [28,42,43], the affinity for the human histamine H3R was tested utilizing radioligand displacement assays with [^3^H]*N*^α^-methylhistamine at membrane preparations from human embryonic kidney (HEK)-293 cells, stably expressing the receptor. Assays ran in triplicates with seven concentrations of the test compounds **1**–**16** (Table 1). The analysis of data was conducted by the software GraphPad Prism 7 (San Diego, CA, USA), using the “one-site competition” equation. *K*_i_ values were calculated from the 50 percent of inhibitory concentration (IC_50_) values according to the Cheng-Prusoff equation [44] (Table 1).

#### 2.1.2. Human Histamine H1 Receptor (hH1R) Binding Affinity for Selected Compounds **4**, **7** and **13**

Radioligand binding was performed as previously described using membranes from CHO-K1 cells stably transfected with the human H1 receptor (PerkinElmer., Waltham, MA, USA) [30,42]. Data were fitted to a one-site curve-fitting equation with Prism 6 (GraphPad Software, city, state, country) and *K*_i_ values were estimated from the Cheng–Prusoff equation [44].

#### 2.1.3. Human Histamine H4 Receptor (hH4R) Binding Affinity for Selected Compounds **4**, **7** and **13**

H4R radioligand displacement assays were performed with Sf9 cell membrane preparations, expressing the hH4R, as described previously [28,42,43]. Assays ran in triplicates with four appropriate concentrations of the test compound. Data were analyzed by GraphPad Prism 7, using the “one-site competition” equation.

### 2.2. In Vivo Pharmacology

#### 2.2.1. Animals

Inbred male Wistar rats aged between six and eight weeks (body weight: 180–220 g, Central Animal Facility of the United Arab Emirates (UAE) University, Al Ain/Abu Dhabi, United Arab Emirates) were used. Animals were kept in an air-conditioned animal facility room with controlled temperature (24 ± 1 °C) and humidity (55 ± 15%) under a 12-h light/dark cycle. The animals were allowed free access to food and water. The experiments of the current study were carried out between 09:00 and 12:00, and all procedures were performed according to the guidelines of the European Communities Council Directive of 24 November 1986 (86/609/European Economic Community (EEC)) and were previously approved for epilepsy study by the College of Medicine and Health Sciences/United Arab Emirates University (Institutional Animal Ethics Committee, approval number; ERA_2017_5676).

#### 2.2.2. Drugs

H3R agonist (*R*)-α-methylhistamine (RAMH, 10 mg/kg, i.p.), pentylenetetrazole (PTZ, 60 mg/kg, i.p.), strychnine (STR, 3.5 mg/kg, i.p.), phenytoin (PHT), and valproic acid (VPA, 300 mg/kg, i.p) were purchased from Sigma-Aldrich (St Louis, MI, USA). H3R antagonists: (**1**) 3-methyl-1-(5-(naphthalen-2-yloxy)pentyl)piperidine hydrogen oxalate [45]; (**2**) 1-(5-(naphthalen-2-yloxy)pentyl)azepane hydrogen oxalate [45]; (**3**) 3-methyl-1-(6-(naphthalen-2-yloxy)hexyl)piperidine hydrogen oxalate [45]; (**4**) 1-(6-(naphthalen-2-yloxy)hexyl)azepane hydrogen oxalate [45]. All other compounds are unpublished to best of our knowledge: (**5**) phenyl(4-(6-(piperidin-1-yl)hexyloxy)phenyl)methanone hydrogen oxalate; (**6**) (4-(6-(3-methylpiperidin-1-yl)hexyloxy)phenyl)(phenyl)methanone hydrogen oxalate; (**7**) (4-(6-(azepan-1-yl)hexyloxy)phenyl)(phenyl)methanone hydrogen oxalate; (**8**) phenyl(3-(6-(piperidin-1-yl)hexyloxy)phenyl)methanone hydrogen oxalate (unpublished); (**9**) (3-(6-(3-methylpiperidin-1-yl)hexyloxy)phenyl)(phenyl)methanone hydrogen oxalate; (**10**) (3-(6-(azepan-1-yl)hexyloxy)phenyl)(phenyl)methanone hydrogen oxalate; (**11**) 1-(6-(4-fluorophenoxy)hexyl)piperidine hydrogen oxalate; (**12**) 1-(6-(4-fluorophenoxy)hexyl)-3-methylpiperidine hydrogen oxalate; (**13**) 1-(6-(4-fluorophenoxy)hexyl)azepane hydrogen oxalate; (**14**) phenyl(4-(5-(piperidin-1-yl)pentyloxy)phenyl)methanone hydrogen oxalate; (**15**) (4-(5-(3-methylpiperidin-1-yl)pentyloxy)phenyl)(phenyl)methanone hydrogen oxalate; and (**16**) (4-(5-(azepan-1-yl)pentyloxy)phenyl)(phenyl)methanone hydrogen oxalate. These synthesized by us in the Department of Technology and Biotechnology of Drugs (Kraków, Poland) (Table 1) according to methods described previously [24,45]. For the in vivo anticonvulsant screening, test compounds **1**–**16**, PHT, VPA, and RAMH were suspended in 1% aqueous solution of Tween 80 and administered intraperitoneally (i.p.) at a volume of 1 mL/kg 30–45 min before the test. Control animals (negative control) were given an appropriate amount of vehicle (1% aqueous solution of Tween 80; i.p.) 30–45 min before the test. Doses of all test compounds were expressed in terms of the free base. For each test compound, a group of six to seven animals was used for the anticonvulsant study (Table 1).

#### 2.2.3. Maximal Electroshock (MES)-Induced Seizure

**Figure 1:**
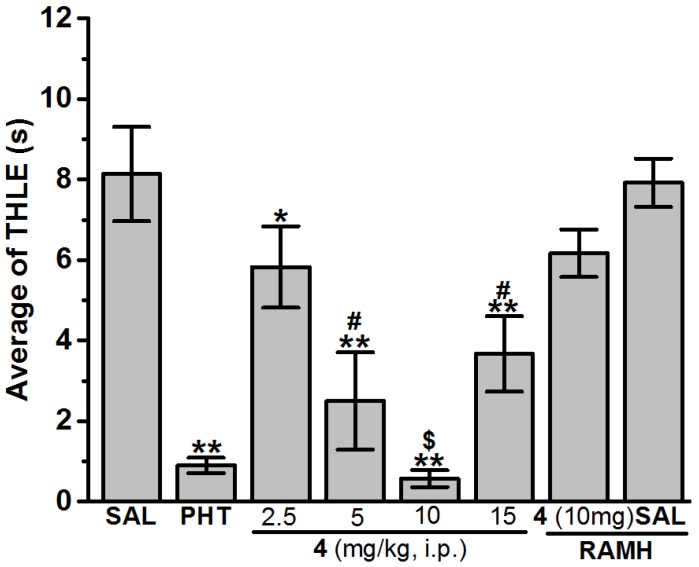
Dose-dependent protective effect of histamine H3 receptor (H3R) antagonist **4** and effect of (*R*)-α-methylhistamine (RAMH) pretreatment on the H3R antagonist **4** provided protection against maximal electroshock (MES)-induced convulsions. The figure shows the protection provided by standard antiepileptic drug (AED) phenytoin (PHT, 10 mg/kg, i.p.) and test compound **4** (2.5, 5, 10, and 15 mg/kg, i.p.) on the duration of tonic hind limb extension (THLE) induced in the MES model in rats. Each value represents mean ± standard error of mean (SEM) (*n* = 6–7). * *p* < 0.05 vs. sal (saline)-treated group. ** *p* < 0.001 vs. (saline)-treated group. ^#^ *p* < 0.05 vs. (2.5 mg)-treated group. ^$^ *p* < 0.05 vs. (5 mg or 15 mg)-treated groups.

As previously described [30,46], the seizures were induced in rats with a 50 Hz alternating current of 120 mA intensity. The current was applied through ear electrodes for 1 s. Protection against the spread of MES-induced seizure was defined as the abolition of the tonic hind limb extension (THLE) component of the seizure [25,29,47]. For the first screening of protective effects of test compounds **1**–**16** in the MES-induced seizure model, the animals were divided into eighteen groups of seven rats (*n* = 7) as follows; group (1) control group injected with vehicle (SAL), group (2) positive control group in which rats were injected with PHT at a dose of 10 mg/kg (this being the minimal dose of PHT that protected animals against the spread of MES-induced seizures without mortality), and groups (3)–(18) animals in the experimental groups were administered H3R antagonists **1**–**16** at a dose of 10 mg/kg. In another MES experiment in three separate groups of six rats (*n* = 6), H3R antagonist **4** with the most promising protection in MES model was administered at doses of 2.5, 5, or 15 mg/kg, i.p. 30–45 min before the MES challenge. In an additional abrogative experiment, the most effective dose of H3R antagonist **4** was designated for further analysis in which a separate group of six rats (*n* = 6) was co-injected with the selected dose of H3R antagonist **4** (30–45 min prior to MES test) with RAMH (10 mg/kg, i.p., 15–20 min before MES challenge) (Figure 1, Table 2).

#### 2.2.4. Chemically-Induced Seizures

In this study and according to previously used protocols, two chemical agents have been used to induce seizures, namely pentylenetetrazole (PTZ, 60 mg/kg, i.p.) and strychnine (STR, 3.5 mg/kg, i.p.) [27,47]. PTZ 60 mg/kg or STR 3.5 mg/kg were administered i.p. to all groups of seven rats (*n* = 7), that is, animals pretreated with vehicle or test compounds **1**–**16**. Vehicle, VPA (300 mg/kg, i.p.), [26,47,48,49] or test compounds **1**–**16** (10 mg/kg, i.p.) were systemically administered 30–45 min before PTZ (60 mg/kg, i.p.) or STR (3.5 mg/kg, i.p.) injection, and animals were directly observed for any signs of convulsion for a duration of 30 min. The observed scores as well as percent protection against generalized tonic-clonic seizure (GTCS) were graded and scaled according to the Racine scale (stage 0, no change in behavior; stage 1, stereotype mouthing, eye blinking, and/or mild facial clonus; stage 2, head nodding and/or severe facial clonus; stage 3, myoclonic jerk in forelimbs; stage 4, clonic convulsions in the forelimbs with rearing; stage 5, generalized colonic convulsions associated with loss of balance [50]) to evaluate the protection provided by the respective test compound against seizures. The animals were divided into eighteen groups of seven rats (*n* = 7) and treated as follows.

**Figure 2:**
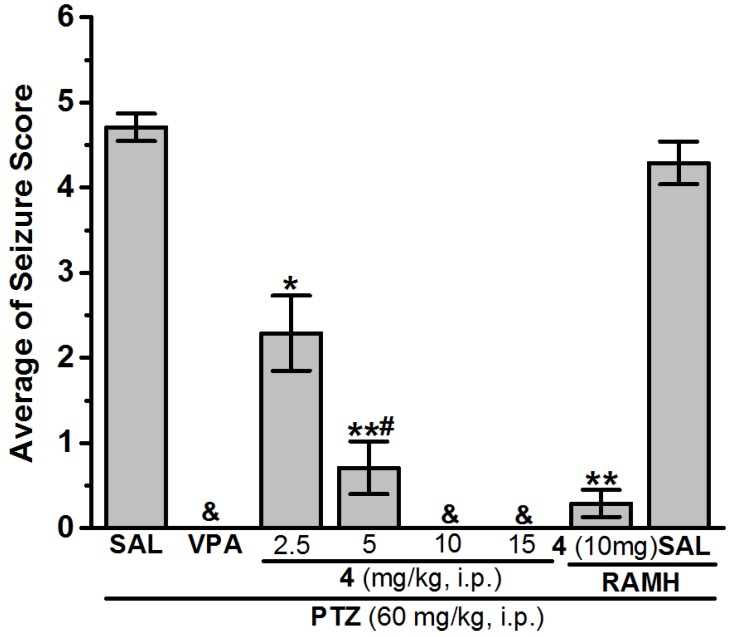
Anticonvulsant effect of H3R antagonist **4** pretreatment on pentylenetetrazole (PTZ)-induced convulsion in rats. Valproic acid (VPA, 300 mg/kg, i.p.) and test compound **4** (2.5, 5, 10, and 15 mg/kg, i.p.) were injected 30–45 min before PTZ (60 mg/kg, i.p.) treatments. Values are represented as the mean ± SEM (*n* = 7). * *p* < 0.05 vs. (saline)-treated group. ** *p* < 0.001 vs. (saline)-treated group. ^#^ *p* < 0.05 vs. (2.5 mg)-treated group. ^&^ Full protection.

**Figure 3:**
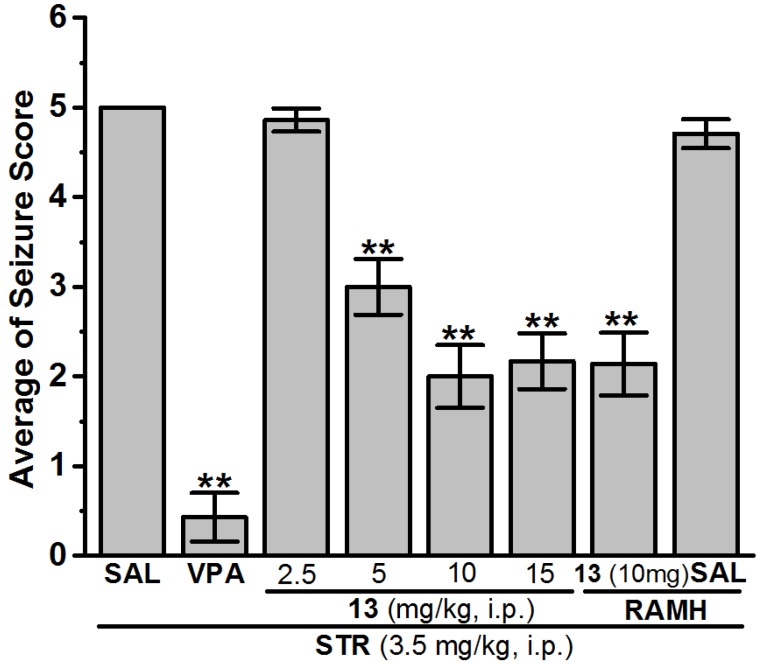
Anticonvulsant effect of H3R antagonist **13** pretreatment on strychnine (STR)-induced seizure in rats. Valproic acid (VPA, 300 mg/kg, i.p.) and test compound **13** (2.5, 5, 10, and 15 mg/kg, i.p.) were injected 30–45 min before STR (3.5 mg/kg, i.p.) treatments. Values are represented as the mean ± SEM (*n* = 7). ** *p* < 0.001 vs. (saline)-treated group.

(1) Control group injected with vehicle + PTZ (SAL group), (2) positive control group in which rats were injected with VPA (300 mg/kg) + PTZ (VPA group), and animals in the test groups (3)–(18) were systemically administered with test compounds **1**–**16** (10 mg/kg), respectively. An additional PTZ experiment was conducted using three different groups of seven rats (*n* = 7), the H3R ligand with the most promising protection in PTZ model was administered at doses of 2.5, 5, or 15 mg/kg, i.p. 30–45 min before the PTZ challenge. In an additional abrogative PTZ experiment, the most effective dose of H3R antagonist **4** was selected for further analysis, namely in a separate group of seven rats (*n* = 7), the selected dose of **4** was co-injected (30–45 min prior to PTZ test) together with RAMH (10 mg/kg, i.p., 15–20 min before PTZ challenge) (Figure 2, Table 2). The same experimental procedure was followed in the STR-induced seizure model applying the reference drug VPA, which was used at a dose of 300 mg/kg, i.p. (Figure 3, Table 2).

#### 2.2.5. Statistical Analysis

For statistical comparisons, the software package SPSS 25.0 (IBM Middle East, Dubai, UAE) was used. All data are expressed as the means ± standard error of mean (SEM). *K*_i_ values at the H3R are given as means with the 95% confidence interval. Following normal distribution assessment, the anticonvulsant effects observed for H3R antagonists **1**–**16** in MES-, PTZ-, and STR-induced convulsion models were analyzed using one-way analysis of variance, followed by the *Bonferroni* post hoc test for multiple comparisons. The criterion for statistical significance was set at *p* < 0.05.

### 2.3. ADME-Tox Properties

#### 2.3.1. Antiproliferative Activity

Human embryonic kidney (HEK)-293 cell line (ATCC CRL-1573) was kindly donated by Prof. Dr. Christa Müller (Pharmaceutical Institute, Pharmaceutical Chemistry I, University of Bonn). *Hepatoma* HepG2 (ATCC HB-8065) cell line was kindly donated by the Department of Pharmacological Screening, Jagiellonian University Medical College. The cell cultures’ growth conditions in presence of H3R antagonist **4** were applied as described before [46,51]. The cells’ viability was assessed after 72 h of incubation with H3R antagonist and the following references: doxorubicin (Sigma-Aldrich) and carbonyl cyanide 3-chlorophenylhydrazone (CCCP) (Sigma-Aldrich). CellTiter 96^®^ AQueous non-radioactive cell proliferation assay (MTS) was purchased from Promega^®^ and added to each well in the volume of 20 µL. The cells were then incubated for 2–5 h. The microplate reader EnSpire (PerkinElmer Ltd., Waltham, MA, USA) was used to measure the absorbance at 490 nm.

#### 2.3.2. Prediction of In Silico Metabolism

The in silico prediction for sites of metabolism of H3R antagonist **4** was performed by MetaSite 5.1.1 provided by Molecular Discovery Ltd. (Borehamwood, Hertfordshire, UK). The most probable sites of metabolism were predicted during this study by the liver computational model [52] (Figure 5).

#### 2.3.3. Metabolic Stability

Rat liver microsomes (RLMs) purchased form Sigma-Aldrich (St. Louis, MO, USA) were used for in vitro determination of compound **4**’s metabolic stability. The NADPH regeneration system was purchased from Promega (Madison, WI, USA). All experiments were performed as described before [26,45,46]. The reaction mixture was preincubated at 37 °C for 5 min, and then the reaction was started by adding 50 µL of NADPH regeneration system. The reaction was ended after 120 min by the addition of cold methanol (200 µL). The mixture was then centrifuged at 14,000 rpm for 15 min and the ultra-performance liquid chromatography–mass spectrometry (UPLC/MS) analysis of the supernatant was conducted. Ion fragment analyses were also performed for metabolic pathways’ determination.

#### 2.3.4. Metabolic Interactions

The metabolic interactions of H3R antagonist **4** were determined by the luminescent P450-Glo™ 3A4 and 2D6 assays purchased from Promega^®^. All enzymatic reactions were performed according to the manufacturer protocols and as previously described [26]. The references ketoconazole (KE, CYP3A4 inhibitor) and quinidine (QD, CYP2D6 inhibitor) were obtained from Sigma-Aldrich. The luminescence was measured with a microplate reader EnSpire (PerkinElmer). The final concentration of H3R antagonist **4** was in the range of 0.1–25 µM, whereas reference inhibitory effects ranged from 0.01 µM to 10 µM. The IC_50_ values’ calculations were performed by GraphPad Prism™ software (version 5.01, San Diego, CA, USA).

## 3. Results

### 3.1. Pharmacology

#### 3.1.1. In Vitro Affinities at hH1Rs, hH3Rs, and hH4Rs

The novel ligands **1**–**16** were tested for their H3R affinity by [^3^H]*N*^α^-methylhistamine displacement assays on membrane preparations of HEK-293 cells, stably expressing the hH3R (Table 1). Following assessment of in vivo anticonvulsant effects in MES-, PTZ-, and STR-induced seizure models for H3R antagonists **1**–**16**, only selected H3R antagonists (**4**, **7**, and **13**), with the most promising in vivo anticonvulsant effects, were further evaluated for their affinity at human histamine H1 (hH1R) and H4 (hH4R) receptors. The results show that test compounds **1**–**16** had an H3R affinity of 40–140 nM compared with the standard H3R antagonist PIT with an H3R affinity of 12 nM (Table 1). Selected test compounds with the most promising in vivo anticonvulsant effects, namely **4** (1273.0 nM for H1R, 69.3 nM for H3R, >10,000 nM for H4R), **7** (915 nM for H1R, 40.5 nM for H3R, >10,000 nM for H4R), and **13** (1338.0 nM for H1R, 137.2 nM for H3R, >10,000 nM for H4R), showed a selectivity profile toward H3Rs with at least 10-fold lower affinity at hH1- and H4Rs.

#### 3.1.2. In Vivo Seizure Models 

With regard to the MES test, the current one used in the study produced seizures in 100% of animals without mortality. Likewise, the dose of PTZ and STR used in the present study formed seizures (score 4–5) in 100% of animals without mortality.

##### Anticonvulsant Screening of H3R Antagonists **1**–**16** in MES-Induced Seizure

The preliminary screening for anticonvulsant activities of acute systemic pretreatment with H3R ligands **1**–**16** on MES-induced seizures in rats was carried out and the observed results were compared with the protective effect of the reference antiepileptic drug PHT in MES-induced seizure in rats (Table 2 and Table 3). The obtained results showed that acute systemic administration of PHT (10 mg/kg, i.p.) and H3R ligands **1**–**16** (10 mg/kg, i.p.) demonstrated a significant protection against MES-induced seizures as confirmed by one-way analysis of variance [*F*_(17,108)_ = 8.352; *p* < 0.001]. Among the H3R antagonists tested and following post hoc analyses, compound **4** at a dose of 10 mg/kg significantly exhibited the most promising protective effect in MES-induced seizure when compared with the saline-treated group with [*F*_(1,12)_ = 34.608; *p* < 0.001], and provided comparable protection to that of PHT with [*F*_(1,12)_ = 1.135; *p* < 0.308] (Table 2 and Table 3, Figure 1). Moreover, the protection observed with H3R antagonist **4** at a dose of 10 mg/kg, i.p. was significantly higher than that found for H3R antagonists **3**, **5**, **6**, **7**, **8**, and **14** with [*F*_(1,12)_ = 5.882; *p* < 0.05], [*F*_(1,12)_ = 9.722; *p* < 0.05], [*F*_(1,12)_ = 8.20; *p* < 0.05], [*F*_(1,12)_ = 6.030; *p* < 0.05], [*F*_(1,12)_ = 11.377; *p* < 0.05], and [*F*_(1,12)_ = 7.108; *p* < 0.05], respectively (Table S1, Supplementary Material). On the other hand, the results show that animals pretreated with 2.5, 5, and 15 mg/kg of H3R antagonist **4** were protected to a significantly lesser extent against seizures when compared with the H3R antagonist **4** (10 mg)-treated group with [*F*_(1,11)_ = 6.087; *p* < 0.05] [*F*_(1,11)_ = 21.843; *p* < 0.001], and [*F*_(1,11)_ = 8.609; *p* < 0.05], respectively (Figure 1). Moreover, the abrogation of H3R antagonist **4**-provided protection was assessed by systemic co-administration with CNS penetrant histamine H3R agonist RAMH (10 mg/kg, i.p.). The results showed that co-injection with CNS penetrant histamine H3R agonist RAMH (10 mg/kg, i.p.) abrogated the H3R antagonist **4** (10 mg)-provided protection with [*F*_(1,10)_ = 0.711; *p* = 0.419] for the comparison of saline–saline vs. **4** + RAMH (Figure 2). Notably, RAMH when administered alone did not affect MES-induced seizures with [*F*_(1,10)_ = 0.359; *p* = 0.563] for saline–saline vs. saline–RAMH (Figure 1).

##### Anticonvulsant Screening for H3R Antagonists **1**–**16** in PTZ-Induced Seizures

The protective effects of H3R antagonists **1**–**16** (10 mg/kg, i.p.) were assessed and compared with the protection obtained for the reference antiepileptic drug VPA in PTZ-induced seizures in rats (Table 2 and Table 3). The results show that acute systemic pretreatment with VPA (300 mg/kg, i.p.) and H3R antagonists **1**–**16** delivered a significant protective action against PTZ-induced seizures as confirmed by applying one-way analysis of variance [*F*_(17,108)_ = 23.925; *p* < 0.001] (Table 2 and Table 3). Pairwise comparison of the provided protective effects observed over 30 min revealed that H3R antagonists **4**, **7**, and **11** delivered full protective activities when compared with the saline-treated group (all *p* < 0.001) (Table 2 and Table 3). Similarly, VPA (300 mg/kg, i.p.) provided full protection when compared with saline-treated group with [*F*_(1,12)_ = 653.40; *p* < 0.001] (Table 2 and Table 3). Moreover, analysis of variance revealed that full protection was provided after acute systemic administration with 10 or 15 mg/kg of H3R antagonist **4** (all *p* < 0.001) (Figure 2, Table 2 and Table 3). However, pretreatment with 2.5 or 5 mg/kg of H3R antagonist **4** provided significantly lower protection when compared with that provided with 10 or 15 mg/kg of the same compound (all *p* < 0.05) (Figure 2, Table 2 and Table 3). Furthermore, Figure 2 shows the reversal of H3R antagonist **4**-provided protection when co-injected with 10 mg/kg of histamine H3R agonist RAMH. The observed results showed that RAMH failed to reverse the H3R antagonist **4**-provided protection in PTZ-induced seizure model with [*F*_(1,12)_ = 2.40; *p* = 0.147] for **6**-treated group versus **4** + RAMH-treated group (Figure 2). Importantly, RAMH alone did not affect seizure score when compared with effects of the saline-treated group with *p* = 0.232 (Figure 2, Table 2 and Table 3 ).

##### Anticonvulsant Screening for H3R Antagonists **1**–**16** in STR-Induced Seizures

In STR-induced seizure in rats, H3R antagonist **4** with the most promising effect in MES- and PTZ-induced seizures failed to provide any appreciable protection in STR-induced seizure when compared with the saline-treated group after 30 min of observation time with [*F*_(1,12)_ = 4.50; *p* = 0.055] (Table 2 and Table 3). However, H3R antagonist **13** without protection in MES-induced seizure and with weak protection in PTZ-induced seizure exhibited a moderate protective effect in STR-induced seizure when compared with the saline-treated group with [*F*_(1,12)_ = 63.00; *p* < 0.001] (Figure 3, Table 3). Notably, the reference antiepileptic drug VPA (VPA 300 mg/kg, i.p.) showed significant protection when compared with saline-treated group after 30 min observation time with [*F*_(1,12)_ = 236.308; *p* < 0.001] (Figure 3, Table 2 and Table 3). Moreover, the results showed that acute systemic pretreatment with H3R antagonist **13** at a lower dose (2.5 mg/kg, i.p.) failed to exhibit protection against STR-induced seizures when compared with the saline-treated group with [*F*_(1,12)_ = 1.00; *p* = 0.337] (Figure 3, Table 2 and Table 3). Furthermore, no significant differences in the protection provided by H3R antagonist **13** (10 mg/kg, i.p.) were observed when 5 or 15 mg/kg of the same compound was administered with [*F*_(1,12)_ = 2.842; *p* = 0.118] [*F*_(1,12)_ = 0.079; *p* = 0.784], respectively (Figure 3, Table 3).

### 3.2. ADME-Tox Properties

#### 3.2.1. Antiproliferative Assay

**Figure 4:**
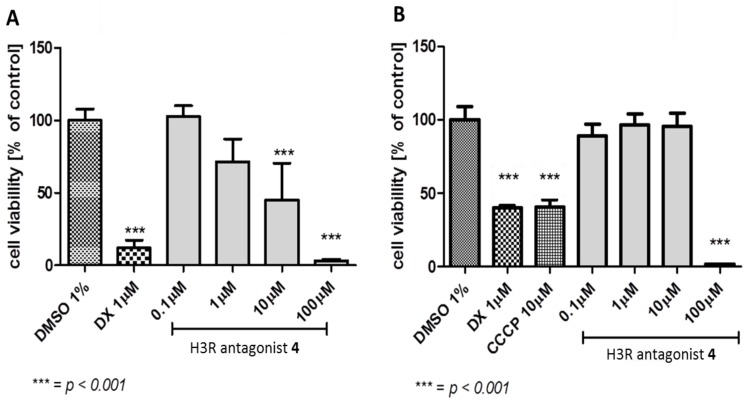
Antiproliferative effects of H3R antagonist **4**. (**A**) Antiproliferative effects of the reference drug doxorubicin (DX) and H3R antagonist **4** on HEK-293 cell line after 72 h of incubation. (**B**) Antiproliferative effects of the reference drug doxorubicin (DX), hepatotoxin carbonyl cyanide 3-chlorophenylhydrazone (CCCP), and H3R antagonist **4** on HepG2 cell line after 72 h of incubation. DMSO: dimethyl sulfoxide.

The effect of H3R antagonist **4** (0.1–100 µM) on proliferation of HEK-293 and *hepatoma* HepG2 cell lines was assessed and compared with the reference cytostatic drug doxorubicin (DX) and hepatotoxin carbonyl cyanide 3-chlorophenylhydrazone (CCCP) (Figure 4A,B). The results showed that H3R antagonist **4** significantly decreased proliferation of HepG2 cells only at the highest concentration used (100 µM), however, decline in proliferation of HEK-293 cells was achieved with two doses of the same compound, namely 10 and 100 µM (*p* < 0.001) (Figure 4A,B).

#### 3.2.2. In Silico Metabolic Stability

**Figure 5:**
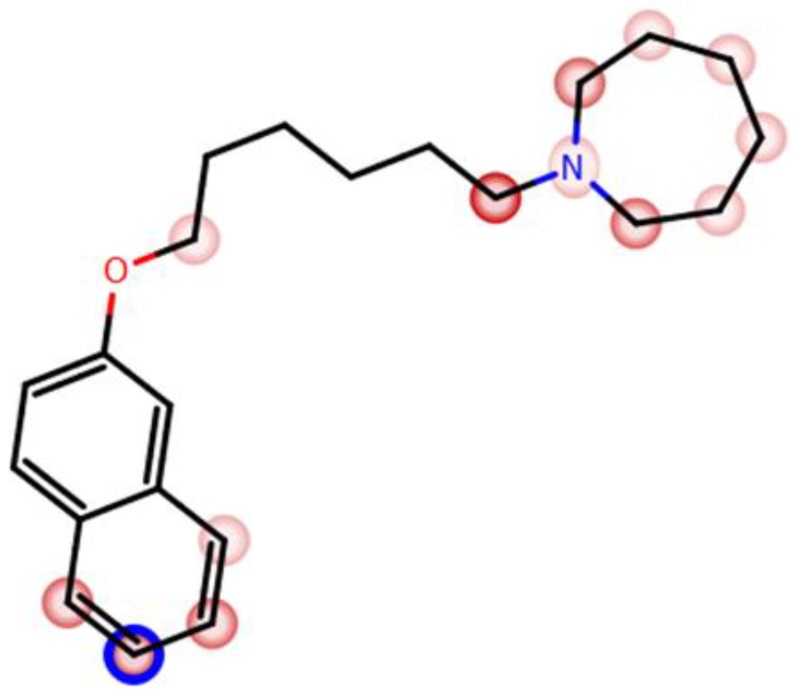
Potential sites of metabolism for H3R antagonist **4**. The blue circle marks the site involved in metabolism with the highest probability. Other potential sites are marked with red color; the darker the color, the higher the probability of involvement in the metabolism pathway (calculated with MetaSite [53]).

The computational procedure MetaSite.5.1.1 provided by Molecular Discovery Ltd. indicated the sixth position of naphthalene moiety as the most probable site of H3R antagonist **4** metabolism (blue circle marked; Figure 5). Moreover, the azepane moiety was also shown to be susceptible for metabolic biotransformations (the darker red color of the marked functional group indicates its higher probability of being involved in the metabolism pathway; Figure 5). The predicted in silico most probable metabolic routes included hydroxylation at the sixth position of naphthalene or azepane group followed by the compound’s oxidative degradation of the aliphatic alkyl chain (data not shown).

#### 3.2.3. In Vitro Metabolic Stability

**Figure 6:**
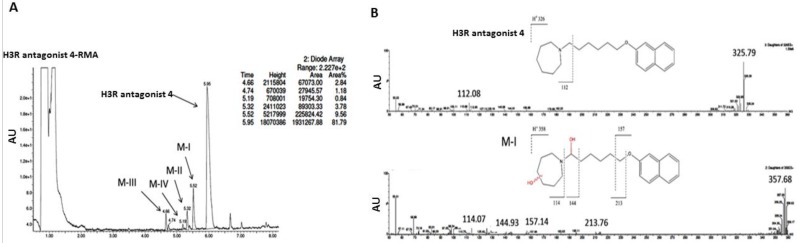

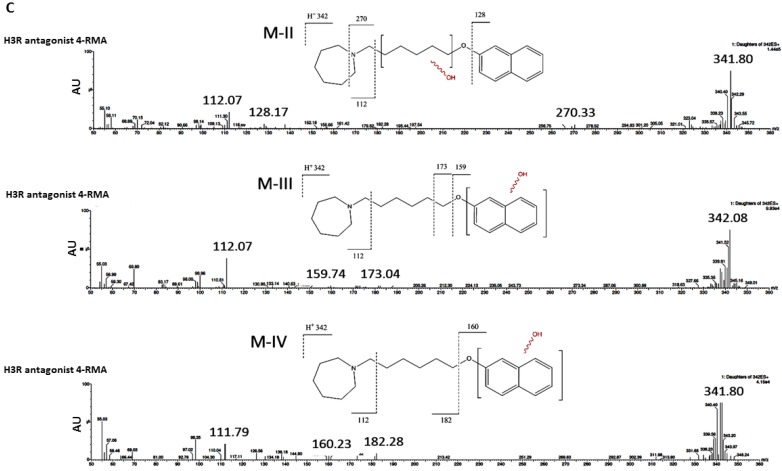
The metabolic profile of H3R antagonist after incubation of H3R antagonist **4** with rat liver microsomes. The UPLC spectrum obtained two hours post incubation of H3R antagonist **6** with rat liver microsomes. (**A**) Around 20% of H3R antagonist **4** was metabolized. (**B**) Main metabolite MI of H3R antagonist **4** with a double-hydroxylation identified as the main metabolic route. (**C**) The mass spectrometry (MS) fragmentation analysis of most probable hydroxylation sites MII–MIV with rat liver microsomes. Abbreviations: UPLC, ultra-performance liquid chromatography.

The UPLC analysis of the reaction mixture of H3R antagonist **4** incubated for 120 min with rat liver microsomes (RLMs) showed that ~18% of this compound was converted into four metabolites, namely M-I–M-IV (Figure 6A). The MS determination of the molecular mass of the main metabolite M-I (+32 units) and the comparison of the ion fragments analyses of H3R antagonist **4** and metabolite M-I suggests the double-hydroxylation at the 6-*n*-hexylazepane moiety as the main metabolic pathway (Figure 6B). Moreover, the hydroxylation reactions at the naphthalene moiety and at the aliphatic alkyl chain were also identified (metabolites M-II–M-IV, Figure 6C). 

#### 3.2.4. Metabolic Interactions 

**Figure 7:**
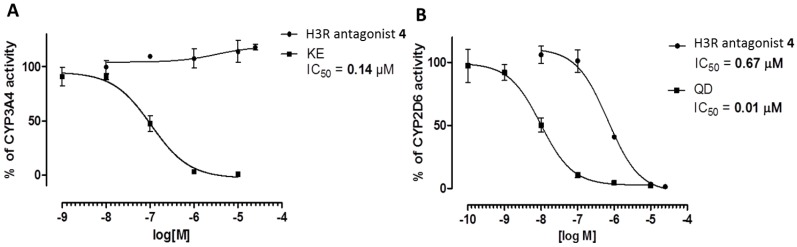
Effect of H3R antagonist **4** on CYP3A4 and CYP2D6 activity. (**A**) Effects of ketoconazole (KE) and H3R antagonist **4** on CYP3A4 activity. (**B**) Effect of quinidine (QD) and H3R antagonist **4** on CYP2D6 activity.

The results observed in luminescent CYP3A4 P450-Glo™ assay showed no inhibitory effects of H3R antagonist **4** on CYP3A4 activity at examined concentrations of 0.1–25 µM (Figure 7A). Moreover, the observed results in luminescent CYP2D6 P450-Glo™ assay revealed that the inhibitory effect of H3R antagonist **6** was detected with an IC_50_ value of 0.67 µM (Figure 7B).

## 4. Discussion

### 4.1. In Vitro Histamine H3 Receptor Affinity of Test Compounds ***1**–**16***

All novel H3R antagonists were evaluated in the form of hydrogen oxalate salts in radioligand displacement assays. Tested H3R antagonists **1**–**16** displayed affinities at the human H3R in a nanomolar concentration range (*K*_i_: 36–137 nM, Table 1). Compounds **13**–**15** displayed *K*_i_ values similar to that of pitolisant (12 nM, Table 1), while the 4-fluorophenyl derivatives were the least potent ones (**11**–**13**; *K*_i_: 83.6–137.2 nM).

### 4.2. Selectivity of Selected H3R Antagonists towards Other Histamine Receptors (H1 and H4)

#### 4.2.1. Histamine H1 Receptor Affinity

As the two histamine receptors H1R and H3R play a pivotal role in the anticonvulsant activity of the central histamine [41], selected H3R antagonists (**6**, **9**, and **15**) were examined in a binding assay at the human histamine H1R and showed weak affinities (915 ≤ *K*_i_ ≤ 1338 nM), being 10-fold lower than at the H3R. 

#### 4.2.2. Histamine H4 Receptor Affinity

As H3R represents the highest degree of homology with H4R [19], the potential interaction with this receptor subtype for selected H3R antagonists (**4**, **7**, and **13**) was evaluated. The observed results showed that none of the tested H3R antagonists displayed affinity for hH4R (*K*_i_ > 10,000 nM). 

### 4.3. In Vivo Anticonvulsant Activity

The results demonstrated that H3R antagonist **4** exhibited the most promising protection against MES-induced seizures when animals were administered with 10 mg/kg i.p., and as compared with the saline-treated group of animals (Table 2 and Table 3). However, lower doses (2.5 and 5 mg/kg, i.p.) and a higher dose (15 mg/kg, i.p.) failed to increase the (10 mg) H3R antagonist **4**-provided protection (Figure 1, Table 3). Accordingly, the observed results show a dose-response relationship of the protection provided and the presence of a ceiling effect for H3R antagonist **4** in the MES-induced seizure model achieved with a dose of 10 mg/kg, i.p. (Figure 1, Table 3). Notably, the protective effect of H3R antagonist **4** (10 mg/kg, i.p.) was similar to that observed in the group treated with the reference antiepileptic drug PHT (10 mg/kg, i.p.), and was significantly higher than that observed for H3R ligands **3**, **5**, **6**, **7**, **8**, and **14** (Figure 1, Table 1). The latter observations are in agreement with recent preclinical outcomes that showed a dose-dependent anticonvulsant effect of several H3R antagonists tested in several animal models of seizures. Also, the present results are in line with earlier observations for the H3R antagonist PIT in a photosensitivity seizure model in adult patients, and in agreement with earlier observations for several H3R antagonists tested for their anticonvulsant potential in different animal models of seizures [25,26,27,28,29,30,33,40,54]. An additional test in the present study showed that the protection observed for H3R antagonist **4** was abolished when animals were co-administered with the CNS penetrant histamine H3R agonist RAMH (10 mg/kg i.p.) (Figure 1, Table 3), proposing that the provided protective effect of H3R antagonist **4** in the MES-induced seizure model involves, at least to some extent, H3R blockade provided by H3R antagonist **4**. Notably, these observations are consistent with the earlier preclinical results for numerous imidazole- and non-imidazole-based H3R antagonists [14,25,26,27,28,29,30,40,54].

H3Rs are auto-receptors presynaptically positioned on histaminergic neurons with an inhibitory effect on the biosynthesis and release of histamine [21]. Therefore, blocking H3Rs by selective H3R antagonists, such as H3R antagonist **4**, would escalate neuronal release of brain histamine, providing the protection in the MES-induced seizure in rats. The latter proposed mechanism underlying the anticonvulsant effect of H3R antagonist **4** is also in line with previous preclinical observations in animal seizure models in which high doses of several centrally acting H1R antagonists used as anti-allergic drugs promoted the development of convulsions of tested animals, indicating the involvement of H1R antagonism, and consequently, brain histaminergic neurotransmission in the seizure promotion. Noticeably, similar protective effects of imidazole-based and non-imidazole-based H3R antagonists were earlier described to be abolished either by H3R agonists or by centrally acting H1R antagonists, suggesting an interaction of the H3R antagonism-released histamine with postsynaptically located H1Rs on neurons [14,25,26,27,28,29,30,53,54].

In the PTZ-induced seizure mode, acute systemic administration of VPA (100 mg/kg, i.p.) as well as H3R antagonists **4**, **7**, and **11** (10 mg/kg, i.p.) showed full protection (Figure 2, Table 3). Furthermore, the anticonvulsant effects observed for H3R antagonist **4** at lower doses (2.5 and 5 mg/kg, i.p.) or a higher dose (15 mg/kg, i.p.) showed a dose-dependent protection in the PTZ-induced seizure model (Figure 2, Table 3). However, the protective effect of H3R antagonist **4** (10 mg/kg, i.p.) was not reversed when rats were pretreated with RAMH (10 mg/kg, i.p.) before PTZ challenge, suggesting that the protection observed for H3R antagonist **4** in PTZ-induced seizure is not facilitated through modulation of central histaminergic neurotransmission (Figure 2, Table 3). The latter observation may be explained with the differences in the triggers or the mechanisms and types of seizures each model represents (MES is considered as a model of generalized tonic-clonic seizures, whereas PTZ (60 mg/kg, i.p.) induces generalized myoclonic and/or tonic-clonic seizures) [11,53,54,55]. The failure of RAMH to reverse the protections provided by H3R antagonist **4** might be explained with its capability to reduce the suppression of glutamatergic and GABAergic synaptic transmission through blockade of H3 heteroreceptor function in CNS, necessitating additional future investigations of whether H3R antagonist **4** modulated the release of inhibitory neurotransmitters, for example, GABA [56].

In the STR-induced seizure model, the results showed that acute systemic administration of H3R antagonists **4**, **7**, and **11** (10 mg/kg, i.p.) failed to exhibit appreciable protection during 30 min of the time observation, whereas H3R antagonist **13** with moderate protection in PTZ and without any considerable protection in MES demonstrated reasonable protection following 30 min during the time observation (Figure 3, Table 3). Moreover, the lower doses (2.5 and 5 mg/kg, i.p.) as well as the higher dose (15 mg/kg, i.p.) of H3R antagonist **13** failed to exhibit a dose-dependent protection against STR-induced seizure in rats (Figure 3, Table 3). In resemblance to the observations in PTZ-induced seizure, an additional experiment showed that the H3R antagonist **13**-provided moderate protection in STR-induced seizure was not reversed when animals were co-injected with 10 mg/kg i.p. of the CNS penetrant histamine H3R agonist RAMH 30–45 min before STR challenge (Figure 3, Table 3). These findings in STR-induced seizure further comprehend the present results for the protective effects of the H3R antagonist **4** in PTZ-induced seizure model, as both models are considered as chemically-induced seizure models. The latter observations for H3R antagonists **1**–**16** in the STR-induced seizure model show that the moderate protection provided with H3R antagonist **13** in STR model is also not facilitated through central histaminergic neurotransmission. Notably, STR is an established competitive antagonist of the inhibitory amino acid glycine. Therefore, the inability of H3R antagonist **13** to afford a dose-dependent protection against STR-induced seizure model advocates little or no modulation effect of H3R antagonist **13** on the glycine receptors, because the mechanisms underlying STR-induced seizures are supposed to be attributed to its blocking activity on glycine receptors in the brain as well as in the spinal cord [56]. Notably, most marketed AEDs were not effective in all conducted convulsion models during preclinical drug development. Accordingly, carbamazepine, oxcarbazepine and PHT were found to be, and are still, highly effective in MES-induced model in rodents, however, they failed to protect against convulsions in rodents induced by PTZ, STR, or picrotoxin [57,58]. On the contrary, ethosuximide and tiagabine, which show high protection in chemically-induced convulsion models in rodents, lack protection in the MES-induced model when used at nontoxic doses [58]. Nonetheless, the diversity in preclinical activities detected for numerous AEDs was translated into the clinical utility of PHT, carbamazepine, and oxcarbazepine, but not ethosuximide or tiagabine, in patients diagnosed with generalized tonic-clonic convulsions.

### 4.4. ADME-Tox Properties

The most promising H3R antagonist **4** was selected to evaluate its ADME-Tox properties, which included the determination of the in vitro safety profile by applying eukaryotic cell lines, the in silico and in vitro determination of the metabolic stability and main metabolic routes, and the in vitro assessment of potential drug-drug interactions by bioluminescent enzymatic assays. 

In order to determine the potential toxicity, the standard colorimetrical MTS test was used. This test allowed one to follow up the influence of H3R antagonist **4** on the human embryonic kidney (HEK-293) and *hepatoma* HepG2 cell lines’ proliferation. The antiproliferative activity of H3R antagonist **4** differed between both used cell lines. HEK-293 cells were more susceptible for antiproliferative activity as revealed in the statistically significant decrease of HEK-293 cell line viability (*p* < 0.001), which was observed at concentrations of 10 and 100 µM, whereas in the HepG2 cell line, the antiproliferative effect was achieved only at a concentration of 100 µM (Figure 4A,B). However, the obtained results for HEK-293 indicated a satisfying safety profile of H3R antagonist **4** in comparison with the reference DX, which significantly decreased HEK-293 viability at a much lower concentration (1 µM) (Figure 4A). Moreover, no significant hepatotoxic effect was observed for H3R antagonist **4**, as the reference drug CCCP decreased HEpG2 viability at 10 µM, whereas H3R antagonist **4** only did so at 100 µM (Figure 4B). 

In further in vitro metabolic stability studies, the results observed for H3R antagonist **4** applying RLMs revealed good metabolic stability of H3R antagonist **4**, as only ~18% of the substrate was metabolized and converted into four different metabolites after 120 min of incubation at 37 °C (Figure 6). Moreover, the in silico data and the MS fragmentation analyses showed one hydroxylation on the azepane moiety and another hydroxylation on the hexyl chain, followed by oxidative degradation of the alkyl chain as the main metabolic routes of H3R antagonist **4** (Figure 5).

Furthermore, H3R antagonist **4** did not affect CYP3A4, whereas CYP2D6 was inhibited in a more pronounced manner. However, this effect was observed with a 67-fold higher IC_50_ (IC_50_ = 0.67 µM) as compared with the reference drug QD with a calculated IC_50_ value of 0.01 µM (Figure 7).

## 5. Conclusions

The tested series of H3R antagonists showed in vitro affinity at the hH3R in the nanomolar range. The most promising H3R antagonist **4** having the 1-(6-(naphthalen-2-yloxy)hexyl)azepane pharmacophore exhibited an affinity for hH3R (*K*_i_ = 69.3 nM). The in vivo anticonvulsant results revealed that H3R antagonist **4** exhibited most promising protection following acute systemic administration in MES- and PTZ-induced seizure models in rats. Moreover, the protection observed for H3R antagonist **4** in the MES-induced seizure model was fully reversed when rats were pretreated with the CNS-penetrant H3R agonist RAMH. However, RAMH failed to abrogate the protective effects observed for H3R antagonist in PTZ- or STR-induced seizure models, indicating that histaminergic pathways appear to be involved in the provided anticonvulsant efficacy of H3R antagonist **4** in only the MES-induced seizure model, but additional pharmacological properties of the compounds or their metabolites cannot be fully excluded. Moreover, ADME-Tox parameters’ screening revealed satisfying low cytotoxicity, good metabolic stability, as well as no inhibition of CYP3A4 activity and moderate inhibition of CYP2D6 activity. Therefore, the overall experimental observations provide promising potential for the novel H3R antagonist **4** to be used as a potential template for further drug design and synthesis in the search for potent and active in vivo H3R antagonists, for example, as AED drugs with a high safety profile. Nonetheless, a battery of additional seizure models with different species is still required to further corroborate the current results observed for H3R antagonist **4**, and to strengthen the translational value of its potential applicability in the therapeutic management of epilepsy.

## Figures and Tables

**Table 1:** In vitro human histamine H3 receptor (hH3R) affinities of ligands **1**–**16**.

Ligand	Structure	In Vitro Affinity *K*_i_ (hH_3_R) ^a^ in nM [CI]
**1**	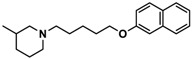	42.3 ^b^ [18.3; 97.4]
**2**	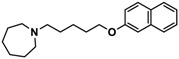	57.3 ^b^ [47.2; 69.7]
**3**	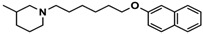	55.9 ^b^ [44.8; 69.7]
**4**	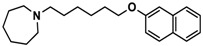	69.3 ^b^ [59.2; 81.1]
**5**	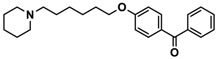	41.1 [25.6; 66.2]
**6**	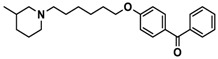	52.8 [31.4; 88.8]
**7**	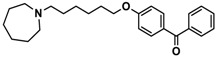	40.5 [32.9; 50.0]
**8**	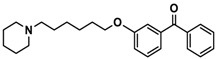	76.1 [53.5; 108.3]
**9**	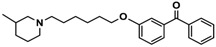	110.2 [61.8; 196.4]
**10**	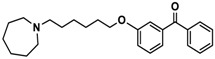	69.5 [44.4; 108.8]
**11**	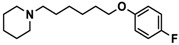	115.2 [78.4; 169.5]
**12**	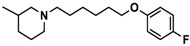	83.6 [65.8; 106.4]
**13**	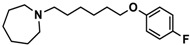	137.2 [60.0; 313.9]
**14**	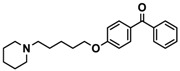	36.2 [10.0; 130.3]
**15**	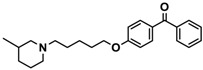	40.2 [13.5; 119.4]
**16**	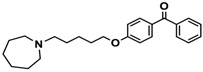	38.5 [10.5; 141.6]
**Pitolisant (PIT)**	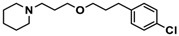	11.69 ^c^

^a^ [^3^H]*N*^α^-Methylhistamine binding assay performed with cell membrane preparations of human embryonic kidney (HEK) cells stably expressing the human histamine H3R. CI—confidence interval; ^b^ data from Łażewska et al., 2018; ^c^ data from the literature [45].

**Table 2:** Screening of in vivo anticonvulsant effects for H3R antagonists **1**–**16**.

Ligand	Structure	MES ^a^-Induced Seizure	PTZ ^b^-Induced Seizure	STR ^c^-Induced Seizure
**1**	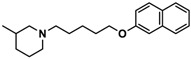	−	−	−
**2**	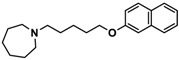	−	−	−
**3**	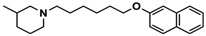	++	−	−
**4**	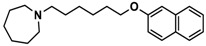	++	++	−
**5**	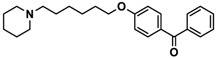	++	−	−
**6**	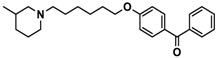	+	−	−
**7**	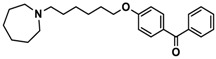	+	++	−
**8**	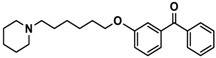	+	−	−
**9**	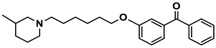	−	−	−
**10**	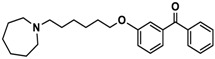	−	−	−
**11**	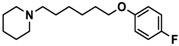	−	++	−
**12**	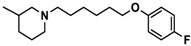	−	+	−
**13**	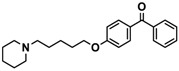	−	+	+
**14**	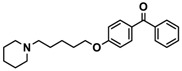	+	+	−
**15**	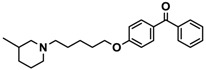	−	+	−
**16**	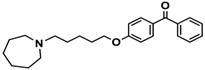	−	−	−

^a^ 50-Hz alternating current of 120 mA intensity applied through ear electrodes for a duration of 1 s, ^b^ 60 mg/kg, ^c^ 3.5 mg/kg. −, no protection (not significant from saline-treated group); +, moderate protective effect (tonic hind limb extension (THLE) duration in the range of 2–5 s, seizure score in the range of 2–4); ++, high protective effect (THLE duration <1, seizure score 0). MES—maximal electroshock; PTZ—pentylenetetrazole; STR—strychnine.

**Table 3:** Effects of H3R antagonists **4** and **13** on THLE duration, average score, and percentage of provided protection against generalized tonic-clonic seizure (GTCS) in MES-, PTZ-, and STR-induced convulsion models.

Group	MES ^a^-Induced Seizure	Group	PTZ ^b^-Induced Seizure		Group	STR ^c^-Induced Seizure	
	Average THLE (s)		Average Seizure Score	% Protection against GTCS		Average Seizure Score	% Protection against GTCS
SAL	8.14 ± 1.17	SAL	4.71 ± 0.16	28.57	SAL	5	0
PHT (10 mg)	0.90 ± 0.19 **	VPA (300 mg)	0.00 ± 0.00	100	VPA (300 mg)	0	100
**4** (2.5 mg)	5.83 ± 1.01 *	**4** (2.5 mg)	2.29 ± 0.44 *	100	**4** (2.5 mg)	−	−
**4** (5 mg)	2.50 ± 1.21 **^,#^	**4** (5 mg)	0.71 ± 0.31 **^,#^	100	**4** (5 mg)	−	−
**4** (10 mg)	0.57 ± 0.21 **^,$^	**4** (10 mg)	0.00 ± 0.00 ^&^	100	**4** (10 mg)	4.57 ± 0.19	28.57
**4** (15 mg)	3.67 ± 0.94 **^,#^	**4** (15 mg)	0.00 ± 0.00 ^&^	100	**4** (15 mg)	−	−
**4** (10 mg) + RAMH	6.17 ± 0.59	**4** (10 mg) + RAMH	0.29 ± 0.16 **	100	**4** (10 mg) + RAMH	−	−
SAL + RAMH	7.92 ± 0.60	SAL + RAMH	4.29 ± 0.25	14.29	SAL + RAMH	−	−
**13** (2.5 mg)	−	**13** (2.5 mg)	−	−	**13** (2.5 mg)	4.86 ± 0.13	14.29
**13** (5 mg)	−	**13** (5 mg)	−	−	**13** (5 mg)	3.00 ± 0.31 **	85.71
**13** (10 mg)	6.43 ± 1.14	**13** (10 mg)	2.14 ± 0.47 *	85.71	**13** (10 mg)	2.00 ± 0.35 **	100
**13** (15 mg)	−	**13** (15 mg)	−	−	**13** (15 mg)	2.17 ± 0.31 **	100
**13** (10 mg) + RAMH	−	**13** (10 mg) + RAMH	−	−	**13** (10 mg) + RAMH	2.14 ± 0.35 **	100
SAL + RAMH	−	SAL + RAMH	−	−	SAL + RAMH	4.71 ± 0.16	28.57

^a^ 50-Hz alternating current of 120 mA intensity applied through ear electrodes for a duration of 1 s,^. b^ 60 mg/kg, ^c^ 3.5 mg/kg. −: not determined. **+**: means that two compounds were co-administered to the tested animals. * *p* < 0.05 vs. (saline)-treated group. ** *p* < 0.001 vs. (saline)-treated group. ^#^ *p* < 0.05 vs. (2.5 mg)-treated group. ^$^ *p* < 0.05 vs. (5 mg or 15 mg)-treated groups. ^&^ Full protection. SAL—saline; PHT—phenytoin; VPA—valproic acid; RAHM—(*R*)-α-methylhistamine.

## Supplementary Materials

Supplementary Materials can be found at http://www.mdpi.com/1422-0067/19/11/3386/s1.
