# Supplementary material for: Studies on Anticonvulsant Effects of Novel Histamine H3R Antagonists in Electrically and Chemically Induced Seizures in Rats
Source: Int J Mol Sci. 2018 Oct 29;19(11):3386. doi: 10.3390/ijms19113386 (PMC6274786; doi:10.3390/ijms19113386)
Supplement: Supplementary file 1 [file ijms-19-03386-s001.pdf]

Supplementary Material

Studies on anticonvulsant effects of novel histamine H3R antagonists in electrically and chemically induced seizures in rats

Alaa Alachkar[1], Dorota Łażewska[2], Gniewomir Latacz[2], Annika Frank[3], Agata Siwek[4], Annamaria Lubelska[2], Ewelina Honkisz-Orzechowska[2], Jadwiga Handzlik[2], Holger Stark[3], Katarzyna Kieć-Kononowicz[2†], Bassem Sadek[1,*†]

[†]These authors have contributed equally to this work.

[1]Department of Pharmacology & Therapeutics, College of Medicine & Health Sciences, United Arab Emirates University, P.O. Box 17666 Al Ain, United Arab Emirates.
[2]Department of Technology and Biotechnology of Drugs, Faculty of Pharmacy, Jagiellonian University Medical College, 9 Medyczna Street, 30-688 Kraków, Poland.
[3]Institute of Pharmaceutical and Medicinal Chemistry, Heinrich Heine University Düsseldorf, Universitaetsstr. 1, 40225 Duesseldorf, Germany.
[4]Department of Pharmacobiology, Faculty of Pharmacy, Jagiellonian University Medical College, 9 Medyczna Street, 30-688 Kraków, Poland.

* Corresponding address: bassem.sadek@uaeu.ac.ae; Tel + 971 3 7137 512; Fax + 971 3 7672 033

Table. Anticonvulsant effects of H3R antagonists 1-16 in MES-, PTZ-, and STR-induced seizures.

Group	MES[a]-induced seizure	Group	PTZ[b]-induced seizure		Group	STR[c]-induced seizure	
	Average THLE (s)		Average seizure score	% Protection against GTCS		Average seizure score	% Protection against GTCS
**SAL**	8.14±1.17	**SAL**	4.71±0.16	28.57	**SAL**	5.00±0.00	NP
**PHT (10 mg)**	0.90±0.19**	**VPA (300 mg)**	0.00±0.00**	100	**VPA (300 mg)**	0.43±0.27**	100
**1**[d]	6.43±1.14	**1**[d]	4.00±0.35	57.14	**1**[d]	4.43±0.27	42.86
**2**[d]	7.86±0.84	**2**[d]	4.43±0.27	42.86	**2**[d]	4.57±0.19	28.57
**3**[d]	1.29±0.17**	**3**[d]	4.29±0.17	71.43	**3**[d]	4.29±0.39	42.86
**4**[d]	0.57±0.21**	**4**[d]	0.00±0.00**	100	**4**[d]	4.57±0.19	28.57
**5**[d]	1.71±0.26**	**5**[d]	4.43±0.34	28.57	**5**[d]	4.57±0.19	42.86
**6**[d]	3.00±0.75*	**6**[d]	4.14±0.24	71.43	**6**[d]	4.43±0.27	42.86
**7**[d]	2.00±0.49**	**7**[d]	0.00±0.00**	100	**7**[d]	4.86±0.13	14.29
**8**[d]	5.71±1.39*	**8**[d]	4.29±0.33	57.14	**8**[d]	4.43±0.19	42.86
**9**[d]	8.29±1.57	**9**[d]	4.57±0.27	28.57	**9**[d]	4.57±0.19	42.86
**10**[d]	6.43±0.75	**10**[d]	4.43±0.19	57.14	**10**[d]	4.71±0.17	28.57
**11**[d]	8.14±0.62	**11**[d]	0.00±0.00**	100	**11**[d]	4.29±0.33	42.86
**12**[d]	7.86±0.51	**12**[d]	2.86±0.62*	71.43	**12**[d]	4.43±0.27	42.86
**13**[d]	6.43±1.14	**13**[d]	2.14±0.47*	85.71	**13**[d]	2.00±0.35**	100
**14**[d]	3.14±0.59*	**14**[d]	3.71±0.56*	42.86	**14**[d]	4.57±0.19	28.57
**15**[d]	7.14±0.74	**15**[d]	3.86±0.51*	57.14	**15**[d]	4.00±0.40	57.14
**16**[d]	6.57±1.22	**16**[d]	4.00±0.53	42.86	**16**[d]	4.43±0.27	42.86
**4**[e]	5.83±1.01$^{*\#}$	**4**[e]	2.29±0.44$^{*\#}$	100	**13**[e]	4.86±0.13	14.29
**4**[f]	2.50±1.21$^{**\#}$	**4**[f]	0.71±0.31$^{**\#}$	100	**13**[f]	3.00±0.31**	85.71
**4**[g]	3.67±0.94$^{**\#}$	**4**[g]	0.00±0.00$^{**\#}$	100	**13**[g]	2.17±0.31**	100
**4**[d]**+RAMH**[d]	6.17±0.59	**4**[d]**+RAMH**[d]	0.29±0.16**	100	**13**[d]**+RAMH**[d]	2.14±0.35	100
**SAL+RAMH**[d]	7.92±0.6	**SAL+RAMH**[d]	4.29±0.25*	14.29	**SAL+RAMH**[d]	4.71±0.16	28.57

[a]50-Hz alternating current of 120 mA intensity applied through ear electrodes for a duration of 1 s. [b]60 mg/kg, [c]3.5 mg/kg. [d]10 mg/kg, [e]2.5 mg/kg, [f]5 mg/kg, [g]15 mg/kg, and H3R antagonists **1-16** (10 mg/kg, i.p.) were injected 30-45 min before MES, PTZ (60 mg/kg, i.p) or STR (3.5 mg/kg, i.p.) challenge. The table shows the protective effects of phenytoin (PHT, 10 mg/kg, i.p.), VPA (300 mg/kg, i.p.) and H3R antagonists **1-16** (10 mg/kg, i.p.) on the duration of tonic hind limb extension (THLE) induced in the maximal electroshock

(MES) model in rats. Protective effects in PTZ- and STR-induced convulsion model are expressed as score of seizures for 30 min observation time after PTZ or STR injection, and percentage generalized tonic-clonic seizures (GTCS) was observed. Dose-dependent effect of H3R antagonist **4** (2.5, 5, 10, and 15 mg/kg, i.p.) on duration of THLE induced in MES-model in rats. Dose-dependent effect of H3R antagonist **13** (2.5, 5, 10, and 15 mg/kg, i.p.) on seizure score as well as percentage of GTCS in STR-model in rats. Effect of RAMH (10 mg/kg, i.p.) pretreatment on the protection provided by H3R antagonist **4** (10 mg/kg, i.p.) against MES- and PTZ-induced seizures. Effect of RAMH (10 mg/kg, i.p.) pretreatment on the protection provided by H3R antagonist **13** (10 mg/kg, i.p.) against STR-induced seizures. Each value represents mean ± SEM (n=6-7). $^{*}P < 0.05$ vs. (saline)-treated group. $^{**}P < 0.001$ vs. (saline)-treated group. $^{\#}P < 0.05$ vs. (5 mg)-treated group.
